# Microwave Hybrid Sintering and Soldering of Cu-Cr-W Composite Material for Reactive Power Breakers

**DOI:** 10.3390/ma17184648

**Published:** 2024-09-22

**Authors:** Sorin Vasile Savu, Cristian Daniel Ghelsingher, Iulian Ștefan, Nicușor-Alin Sîrbu, Andrei-Angelo Midan, Ilie Dumitru, Ionel Dănuț Savu, Claudiu Nicolicescu, Andrej David

**Affiliations:** 1 Department of Engineering and Management of Technological Systems, Faculty of Mechanics, University of Craiova, 200585 Craiova, Romania; iulian.stefan@edu.ucv.ro (I.Ș.); ionel.savu@edu.ucv.ro (I.D.S.); claudiu.nicolicescu@edu.ucv.ro (C.N.); 2 Doctoral School Academician Radu Voinea, Faculty of Mechanics, University of Craiova, 200585 Craiova, Romania; ghelsingher.cristian.j4s@student.ucv.ro (C.D.G.); midan.andrei.c3i@student.ucv.ro (A.-A.M.); 3 NRDI for Welding and Material Testing, 300222 Timisoara, Romania; asirbu@isim.ro; 4 Department of Automotive, Transport and Industrial Engineering, Faculty of Mechanics, University of Craiova, 200585 Craiova, Romania; ilie.dumitru@edu.ucv.ro; 5 Department of Water Transport, Faculty of Operation and Economics of Transport and Communications, University of Zilina, 01026 Zilina, Slovakia; andrej.david@fpedas.uniza.sk

**Keywords:** soldering, microwave heating, thermal field, sintered material

## Abstract

Over 60% of reported failures for reactive power compensation systems are given for damage to electrical circuit breaker contacts. This paper presents a study on the development of microwave technology for sintering of W–Cu–Cr alloys at 1012 °C for 65 min using 623.38 W microwave power, as well as microwave joining at 231 °C of the W–Cu–Cr composite material on body contact using 475 W microwave power for 55 s. The joined components were subjected to mechanical and electrical tests in accordance with ICE standards to validate the applied technology. Tests of connection–disconnection of the electrical contacts were carried out in accordance with the maximum number of disconnections allowed by the manufacturer (2 cycles/min): 25 s rest time and 5 s operating time under load. The components of the electrical contact after 111237 switches were analyzed under a microscope revealing a reduction of the damaged area by 27% compared with the original contact.

## 1. Introduction

**Figure 1:**
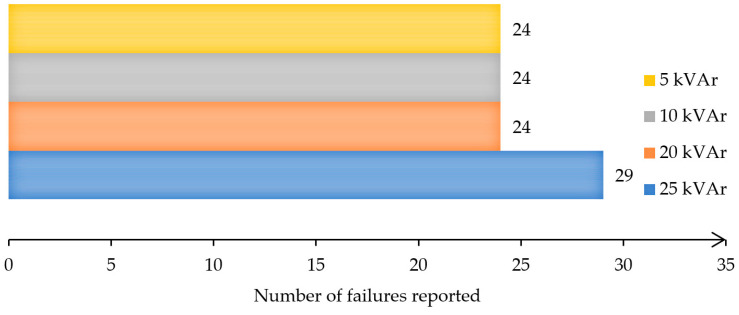
Failures of electrical contacts.

The switchers/breakers intended for connecting/disconnecting capacitive loads are designed and executed with an ambient operating temperature for encapsulated systems in the range of ±40 °C, and a minimum number of operations equal to 10^5^ switches. However, under operating conditions, the active elements of the switching equipment experience overheating of the breaking contacts, thereby leading to their welding [1]. The main negative effect consists in unbalancing the capacitor bank, or not achieving the compensation stage. Moreover, battery imbalance results in an erroneous active/reactive energy measurement when contact welding occurs on only one phase. Failure to achieve the compensation step is directly reflected in the consumer’s final energy costs. According to the annual report of SERTIM Electric [2], the failure to achieve reactive energy compensation is mainly due to the failure of the switching systems, as a result of the destruction of their contacts. Figure 1 presents the number of contact failures in 2023 reported in 800 installed units. According to the report, all systems had been installed at the consumers and had been functioning at normal temperature, humidity, and pressure. The number of failures is an absolute number, as recorded in 2023, and represents the number of breakers replaced after failure.

Microwave sintering is widely applied for ceramics, as well as for metals and composite materials. Dwivedi et al. [3] reported that the synergistic performance evaluation of copper–WC–graphene composites produced via microwave sintering has yielded promising results across various aspects. A microstructural analysis demonstrated a uniform distribution of reinforcing elements (WC and graphene) in the copper matrix for all compositions, highlighting the efficiency of the microwave sintering technique. Sun et al. [4] concluded that microwave hot press sintering led to a notable enhancement in the physical and mechanical properties of the composites, including their density and hardness. In addition, the graphite/copper composites exhibited both excellent thermal conductivity and frictional properties. Wang et al. [5] studied the thermal properties of graphite/copper composite material sintered in a microwave field. They reported that, with the increase in the number of thermal cycles, the thermal diffusion coefficient of the Gr/Cu composites decreased and gradually stabilized. By contrast, the CTE of the composites showed a linear increasing trend. Xu et al. [6] reported on the excellent performance of a W–Cu alloy sintered in a microwave field. The results obtained revealed a more uniform and compact microstructure with high density. Sustainable microwave processing has been reported by Kaushal et al. [7]. They concluded that processing materials/powders through microwave heating is a challenging task; yet, it is promising owing to its exceptional rapid heating characteristics, coupled with its potential energy savings and sustainability nature. The composite castings of Cu–W_x_-based metallic powders were successfully processed through MHH at 2.45 GHz and 900 W. Cursaru et al. [8] studied the sintering process of ceramics for biomechanical applications, referring to microwave irradiation as a sustainable technology for materials sintering. The microwave sintering of materials has limitations, mainly relating to process stability. Stefan et al. [9] studied the thermal runaway phenomenon during the microwave heating of composite materials. They reported that ceramic materials tend to suffer from thermal runaway after 600 °C; this was the case even with low levels of microwave-injected power. Savu I.D. et al. [10] and Savu S.V. et al. [11] concluded that thermal runaway can be avoided by applying a simultaneous reduction of microwave-injected power down to 630 W and a manual control of the matching load impedance to increase the reflection of microwaves but ensuring, at the same time, the protection of the generator antenna. Thermal runaway has been studied widely [12,13,14], and is considered the most influential factor that creates instability during microwave heating.

In addition to microwave sintering, fabrication processes, such as welding, bonding and soldering, can be applied in a microwave field. Zhang et al. [15] investigated the heat transfer from ceramic susceptor to copper materials with SAC305 solder in microwave hybrid soldering. They concluded that cylindrical susceptors provide a more efficient and consistent heating effect compared to cubic susceptors. In addition, when the susceptor’s height is 30 mm, the heating efficiency peaks, and the hot spot is located at the bottom of the susceptor. Said et al. [16] performed a review of microwave hybrid heating (MHH) providing understanding of MHH for an alternative soldering approach by compiling all the most recent research, technical setup, and key discoveries. The MHH soldering method shows remarkable performance for improving the strength (44.4%) of the solder joint compared to the conventional method. Savu S.V. et al. [17] studied the microwave soldering of copper plates for 600, 650, 700, and 750 W at 2450 MHz for a soldering time up to 60 s. They concluded that microwave soldering does not significantly influence the total electrical resistance which is mandatory for electronic applications. Other researchers have studied the grain orientation and failure mechanism of isothermal aged Cu–Cu joints using microwave hybrid heating [18]. Zhang et al. [19] reported that the interface of the MHH joints was larger than that of the reflow soldering joints obtained under the parameters of 195 s at a power of 900 W for process parameters at a peak temperature of 250 °C and a reflow time of 600 s.

This research aims to apply microwave technology for joining W–Cu–Cr composite material and contact body material using hybrid microwave heating. This study is also focused on microwave joining process stability, as well as testing the switching devices according to ICE standards to validate the joining technology and performance of the final product.

## 2. Materials and Methods

To develop the composite material, a mass composition (percentage by weight) of 94% Cu, 1% Cr, and 5% W was used. The powders used for the development of the composite material were as follows: copper powders, produced by Pometon S.p.A, Maerne, Italy; tungsten powders, produced by Electroputere Craiova SA, Romania; and chromium powders, produced by Merck KGaA Sigma Aldrich, Germany. The powders were weighed using an analytical balance: Partner WPS 510/C2, produced by Partner Corporation (London, United Kindom, 2008). The characteristics of the elemental powders are presented in Table 1.

These powders were mechanically alloyed using a planetary ball mill: Pulverisette 4, manufactured by Fritch (Idar-Oberstein, Germany, 2010). The technological parameters of the mechanical alloying process are presented in Table 2.

### 2.1. Modelling and Simulation of Temperature Distribution at Microwave Processing

**Figure 2:**
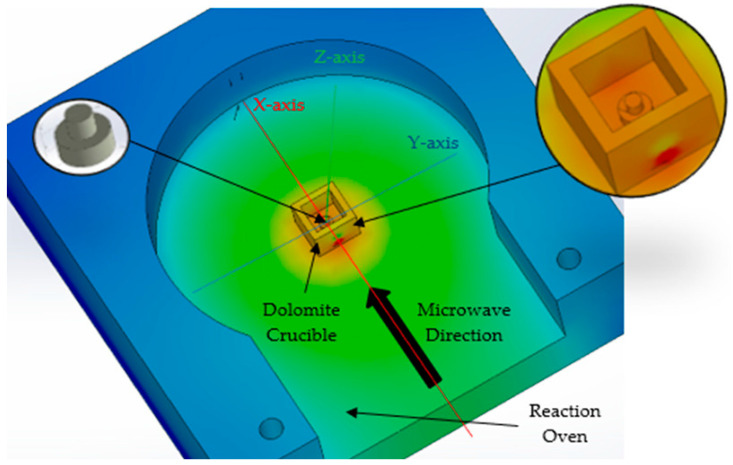
The model developed for temperature distribution on microwave indirect heating.

Microwave sintering of metal-based composite materials cannot be performed due to the high reflection of microwaves by metals [20]. Therefore, indirect microwave heating or hybrid microwave heating can be applied, but only after a simulation of temperature distribution to establish the lowest level of microwave power. Due to the reflection coefficient of metal, high levels of microwave power will lead to a plasma arc discharge and damage of the magnetron antenna. Therefore, to achieve a better conversion rate of the microwaves into heat, ceramic susceptors must be used. This approach defines the indirect or hybrid microwave heating, and usually is taken into consideration for the sintering of materials with a high reflection of microwaves. The heat developed by ceramic susceptors will be transferred based on Fourier’s heat conduction law, as presented in Equation (1):

(1) $$Q=-k\cdot A\cdot \frac{\Delta T}{h}=-k\cdot A\cdot \frac{\left(T_{h}-T_{c}\right)}{\left(h_{e}+h_{i}\right)}$$

where A (mm^2^) is the heat transfer area, T_h_ (°C) is the highest temperature obtained from the conversion of the microwaves into heat, T_c_ = 25 °C is the ambient temperature considered for simulation, and h = h_e_ + h_i_ = 3.22 mm represents the total length of the assembly and k (W/mK) represents thermal conductivity. Figure 2 presents the temperature distribution during microwave hybrid heating.

**Figure 3:**
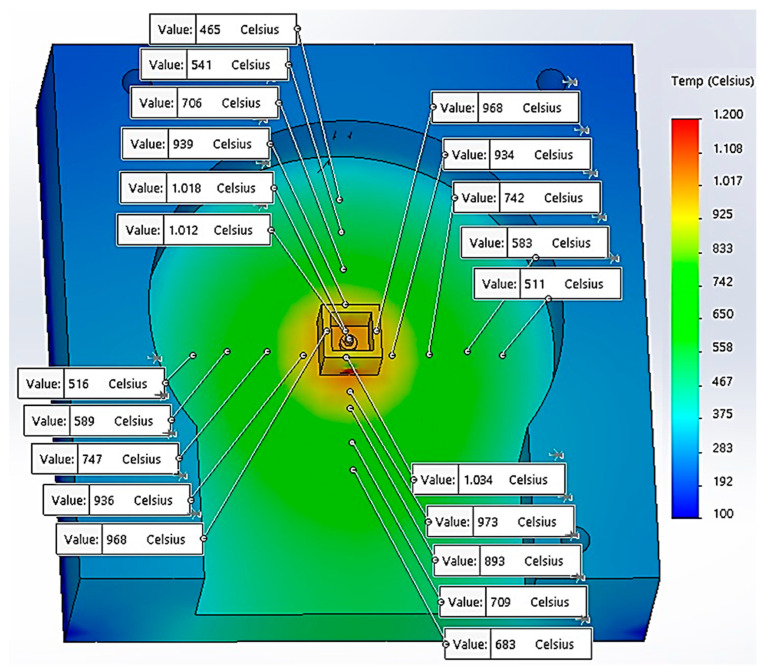
Temperature distribution on Y-axis and X-axis.

The model contains the microwave reaction chamber and a ceramic crucible (dolomite material) with overall dimensions 14 × 14 × 10 mm having a 1 mm-thick wall. The composite material, CuCr1W5, has a specific shape for active elements of the reactive switching devices, consisting of two cylinders with different diameters and heights, as follows: d_e_ = 4 mm, h_e_ = 1.60 mm; and d_i_ = 2 mm, h_i_ = 1.62 mm. Considering the simulation after the *X*-axis and *Y*-axis, the distribution of the temperatures are presented in Figure 3. The simulation has considered the following parameters and boundary conditions related to the heat transfer into solids: the heat conduction on the *Z*-axis is higher towards the bottom of the reaction oven, taking into consideration the contact between the dolomite crucible and the aluminum body of the oven. The heat transfer from the dolomite (indirect microwave heating) to the composite material CuCr1W5 and the reaction oven body uses specific categories of thermal parameters, such as thermal conductivity (k), heat expansion coefficient (α), and specific heat (c), is presented in Table 3 [21,22].

According to the simulation of thermal transient for 5 min, in Figure 3, the temperature distribution, at the contact between the dolomite crucible and the oven body was approximately 930 °C for both the *X*- and *Y*-axis, suggesting a uniform distribution inside the crucible. Similarly, on the *Z*-axis, the simulation of temperature reveals that at the top of the sample the value obtained was 1012 °C.

**Figure 4:**
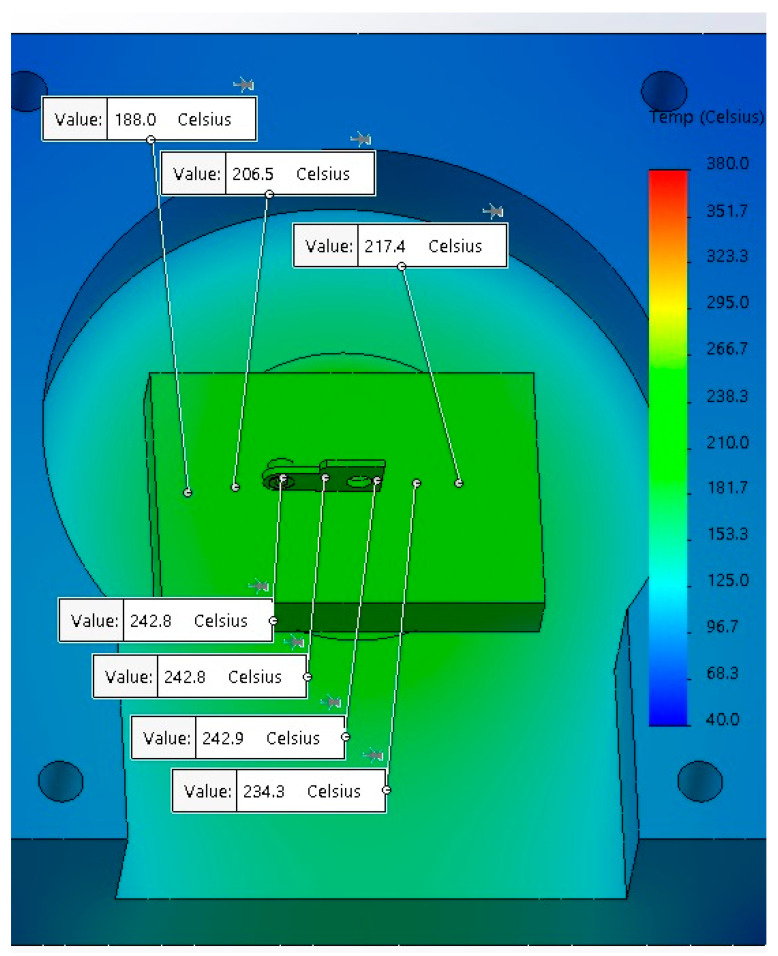
Temperature distribution during microwave soldering process.

The microwave soldering of copper–copper plates has been studied in previous research [17]. The results have shown that various levels of microwave-injected power from 650 W to 750 W can be applied to achieve a stable soldering process in a microwave field with good quality of joints, and with a low, indirect carbon footprint and costs. However, the microwave soldering of composite sintered material based on CuCr1W5 with a contact body requires study on the microwave parameters to achieve a uniform distribution of temperature in the soldering area. Therefore, before soldering in a microwave field, a simulation of temperature distribution is required. That must be studied due to the high reflection coefficient of microwaves of metals. Similarly to microwave sintering, for a joining application, it is necessary to use ceramic susceptors in order to convert microwaves into heat. The model for the simulation of temperature distribution, presented in Figure 4, was performed for a contact body having 15.70 × 10.50 mm dimensions; an active element having d_e_ = 4 mm, h_e_ = 1.60 mm and d_i_ = 2 mm, h_i_ = 1.62 mm; a solder SAC305, with chemical composition SnAg3Cu0.5 produced by Cynel Unipress (Warsaw, Poland, 2023), deposited on contact surfaces; and a dolomite susceptor, with a parallelepiped shape having the overall dimensions 50 × 50 × 40 mm.

The active element of the electrical contact was designed and executed exactly like an industrial contact existing in a reactive switch, type CEM 5 kVAr, produced by ETI Elektroelement d.o.o. (Izlake, Sloven Republic, 2024).

### 2.2. Microwave Sintering of Composite Material

Based on the simulation results, the temperature developed inside the ceramic susceptor (dolomite crucible) was evaluated to be approximately 1013 °C; this will be used to determine the necessary microwave-injected power using the following Equation (2):

(2) $$P_{MW}=k\cdot V\cdot f^{2}\cdot \varepsilon \cdot \tan\delta \cdot \frac{\Delta T}{t}$$

where P_MW_ (W) is the microwave-injected power from the generator, k = 1.01 × 10^−9^ is the proportionality constant depending on the geometry and material properties of the crucible [23], V = 0.0000098 m^3^ is the overall volume of dolomite crucible, f = 2450 MHz is the frequency of microwave radiation, ε = 7.2 F/m [24] is the permittivity of dolomites, tan δ = 4.32 × 10^−4^ at 2450 MHz [25] is loss tangent of dolomites, ΔT = 1012 °C is the temperature required at the contact between crucible and sample material, and t = 300 s is the total time required for reaching the sintering temperature of the composite material. By solving Equation (2), the minimum level of injected power has been determined to be P_MW_ = 623.38 W.

**Figure 5:**
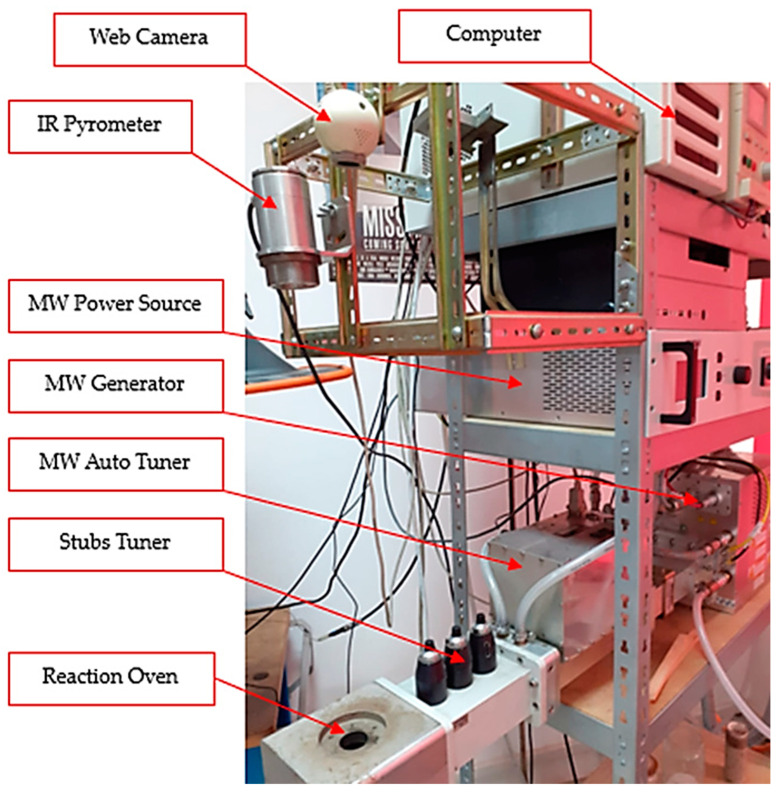
Experimental microwave soldering installation.

By using the calculated microwave power, the experimental program for sintering the composite material based on CuCr1W5 was performed using a microwave installation, as presented in Figure 5.

The microwave sintering procedure of the composite material was supported by a microwave heating installation consisting of an MW-Generator Set 6000 W, 2450 MHz continuous wave containing a magnetron head type MH6000S-251BF, from Muegge GmbH (Reichelsheim, Germany, 2020). The microwave generator was driven by an MW-Power Source Supply 6000 W, 2450 MHz, 3 × 400 V, continuous wave type MX6000D-154KL, both manufactured by Muegge GmbH (Germany, 2020). The main auxiliary devices were an infrared pyrometer CSmicro 3M manufactured by Optris GmbH (Berlin, Germany, 2020), having a temperature range between 50 °C to 600 °C, with a spectral range of 2.3 µm, used for temperatures below 600 °C; and a CT Glass G5H manufactured by Optris GmbH (Germany, 2012), having a temperature range between 250 °C to 1650 °C, with a spectral range of 2.1 µm, used for temperatures above 600 °C. Both devices are ideal for the temperature measurement of metallic surfaces. The control of the infrared pyrometer was performed using a Compact Connect supplied by the manufacturer together with an IR pyrometer. In addition, for matching the load impedance, a Tristan auto tuner up to 6000 W, 2450 MHz, from Muegge GmbH (Reichelsheim, Germany, 2008), was used. The tunning process, related to matching the load impedance, was supported by Homer Software, provided by S-Team d.o.o. (Bratislava, Slovakia, 2008).

**Figure 6:**
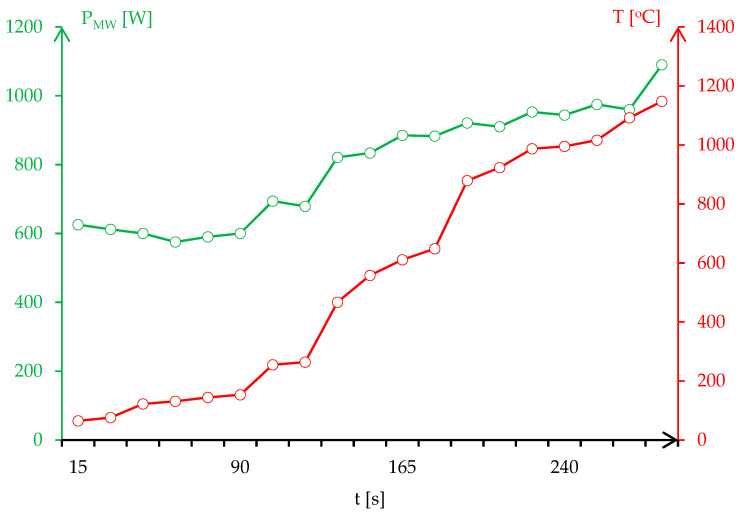
Temperature evolution on the surface of the composite material as a function of the injected power.

The microwave process parameters related to matching the load impedance to the microwave generator, in addition to the injected power, was determined experimentally through the microwave heating of the dolomite crucible without any loads. The values of the stub tuners were calculated and then recorded using the Tristan auto tuner and Homer Software. The process was set for high temperatures up to 1400 °C to be able to analyze the behavior of dolomite in a microwave field without injected powers higher than 600 W. The microwave sintering was performed in a normal atmosphere, but a jet of inert gas (argon) was directed to the samples with a flow rate of 2 L/min. Figure 6 presents the temperature evolution in correlation with the injected power for the resonant circuit.

Once the presumed sintering temperature T = 1012 °C had been reached, the heating process was maintained for 60 min. The sintered sample was then prepared for microscopy analysis.

### 2.3. Hybrid Soldering of Sintered Active Element to the Contact Body

**Figure 7:**
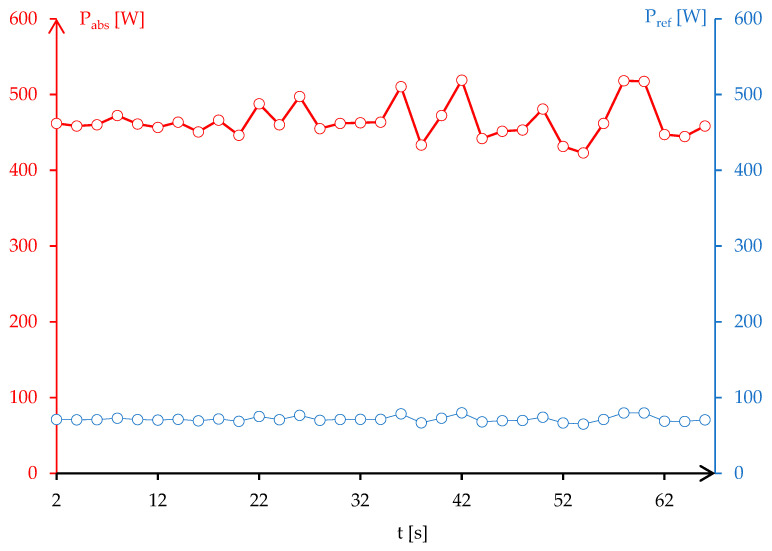
Resonant circuit for microwave soldering of sample to the contact body.

Based on a simulation of the temperature distribution, the necessary microwave-injected power for soldering was calculated using Equation (2) to 533.25 W, taking into consideration the solder SAC305 having the thickness 0.1 mm. This level of power was reached in an experimental application after the calculation of the distance of stub tuners using Equation (3):

(3) $$d_{i}=-\frac{\lambda }{2\pi }\cdot {tan}^{-1}(B_{i}\cdot Z_{0})$$

where d_1_, d_2_, and d_3_ (mm) represent the length of the three stub tuners inside the WR340 waveguide, λ = 0.122 m is the wavelength of microwaves at 2450 MHz, the susceptance of stub tuners being considered B_1_ = B_2_ = B_3_ = 0.5 mS, and Z_0_ = 376.7 Ω is the air impedance. The computation of Equation (3) for the assembly of the susceptor-contact-solder-sample led to the following values: d_1_ = 0 mm, d_2_ = 22.62 mm, and d_3_ = 24.84 mm. The results for stub tuners was validated by the stubbing scenario introduced in Homer Software for manual tunning of microwave heating process. Figure 7 presents the stub tuner movements for resonant circuit and the absorbed and reflected power during the microwave soldering process.

The experimental process of microwave soldering was started using 533 W representing 8.83% of the total available power that can be supplied by a microwave generator. At this level of power, the process tends to be unstable and difficult to control. However, the stability of dolomites in the microwave field led to a rapid increase in temperature. After 55 s, the temperature recorded on the surface of the sample was 231 °C, enough to perform the joining of the sample to the contact body. This level of temperature was obtained at 475 W, according to a three-stub tuner position and the microwave power absorbed, which was lower with 12% of the calculated microwave power using Equation (3). The difference can be explained due to the presence of SAC305 that became liquid after 217 °C, which improved the heating mechanism conditions and led to the better conversion of microwaves into heat.

### 2.4. Testing New Electrical Contact

**Figure 8:**
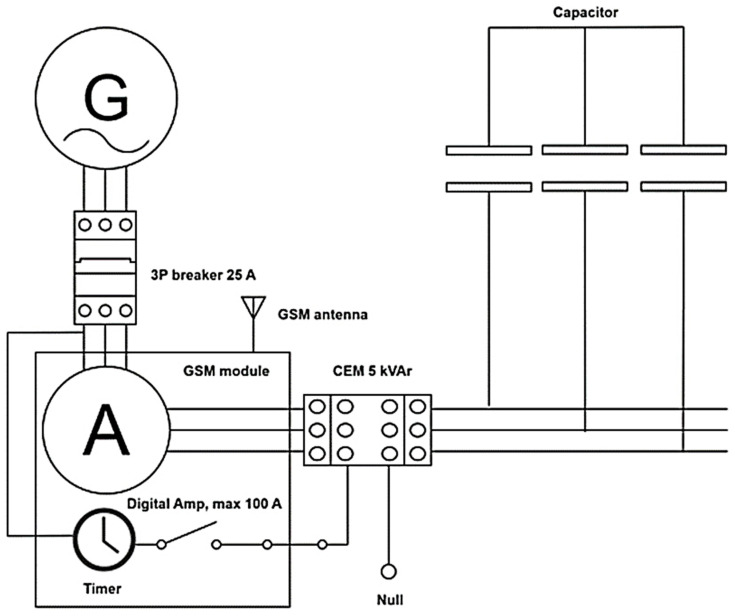
Schematic diagram of experimental device designed for breaking test.

The new electrical contact developed through the microwave sintering of composite material based on CuCr1W5 and soldered in a microwave field to the contact body was tested according to ICE standards for more than 100,000 cycles of breaking reactive circuit. In order to monitor the performance of the breaking device, a system composed of a three-phase reactive capacitor, a digital multimeter for reactive current measurement, a GSM module for the transmission of data measured and the status of the breaker, as well as an integrated circuit for time record has been developed. Figure 8 presents the schematic diagram of the experimental device.

**Figure 9:**
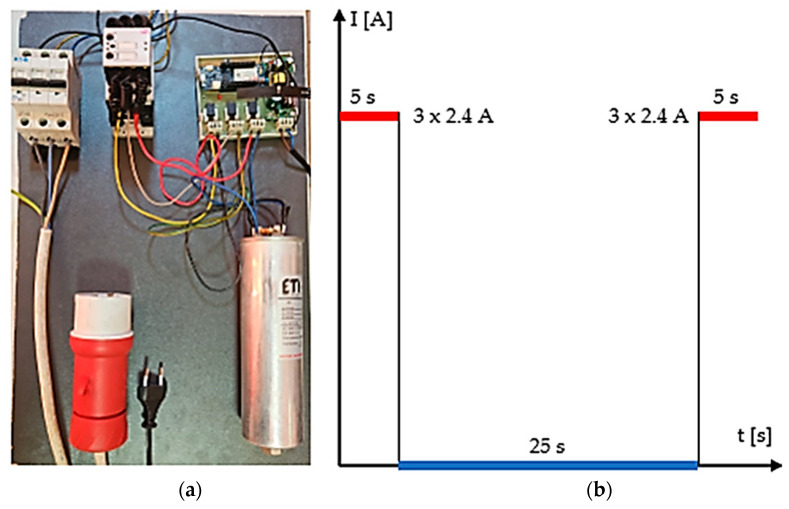
Experimental program for testing the new contact: (**a**) experimental device, (**b**) breaking cycle.

An experimental device has been designed to test the functionality and performance of the new contact for breaking reactive loads. The system consists of a main breaker type 3P PL6–25/3, produced by Eaton (Kearney, NE, USA, 2024), connected to an electrical grid having a nominal breaking current up to 25 A. The main part of the system is composed of a digital multimeter for the measurement of AC currents up to 100 A, using Hall sensors ACS758LCB-050U-PFF-T produced by Allegro MicroSystems (Musselburgh, United Kingdom, 2024), a timer for breaking/connecting cycles integrated, and a GSM module connected to an external antenna both being built in a development motherboard type MKR 1400 GSM manufactured by Arduino LLC (Firenze, Italy, 2024). The functionality of the system is driven by a controller programmed to transmit information in real time in terms of AC current values for each phase. Thus, the breaking circuit CEM 5 kVAr produced by ETI Elektroelement d.o.o. (Izlake, Slovenia, 2024) containing the new contact based on CuCr1W5 will be monitored individually during the breaking cycle. The active part of the system is composed of a breaking circuit and a three-phase capacitor type LPC 3 × 3.33 µF, 5 kVAr, 400 V, 7.2 A connected as a load. Figure 9a presents the manufactured device produced in the scope of the research and Figure 9b presents the breaking/connecting cycle established to evaluate the performance of the new contact.

The testing of functionality and the performance of new electrical contact using composite material based on CuCr1W5 was performed for 111,237 connecting/breaking cycles until the contacts were welded. The information related to the status of the relay contacts was submitted automatically by the MKR 1400 GSM board, manufactured by Arduino LLC (Firenze, Italy, 2024). The monitoring process was not focused only on the failure of the switch/breaker device, but also on the values of the capacitive currents.

The new contact was embedded in the middle (S-phase of 3P system) of the three-phase relay contacts, the other two contacts being originals from the manufacturer. Before embedding the contacts in breakers, the samples were cleaned with alcohol to decontaminate the surface of the active elements. In addition, before installing the contacts into the breaker, the electrical resistance of the samples-contact body was measured using a 4-wire method. The results obtained were as follows: R_R_ = 0.065 Ω for R-phase, R_S_ = 0.055 Ω for S-phase, and R_T_ = 0.060 Ω for T-phase. The capacitive currents measured before failure of the system were as follows: I_R_ = 2.27 A for R-phase, I_S_ = 2.55 A for S-phase, and I_T_ = 2.42 A for T-phase. From the beginning, the system was unbalanced by 11.07% due to the electrical connections and the performance of the capacitor. After 111,237 cycles, the system reported a failure on T-phase due to the welded contacts.

In addition, to evaluate the microhardness of the sintered composite material, a Namicon CV-400 DTS tester produced by CV Instruments (Thornaby, United Kingdom, 2007) was used. The microhardness tests were performed in accordance with EN ISO 6597 standards using Vickers method with the following process parameters: peak angle of indenter—136°, pressing force 4.9 N (HV0.5), and dwell time 15 s. The microhardness results obtained after 3 tests are presented in Table 4.

**Figure 10:**
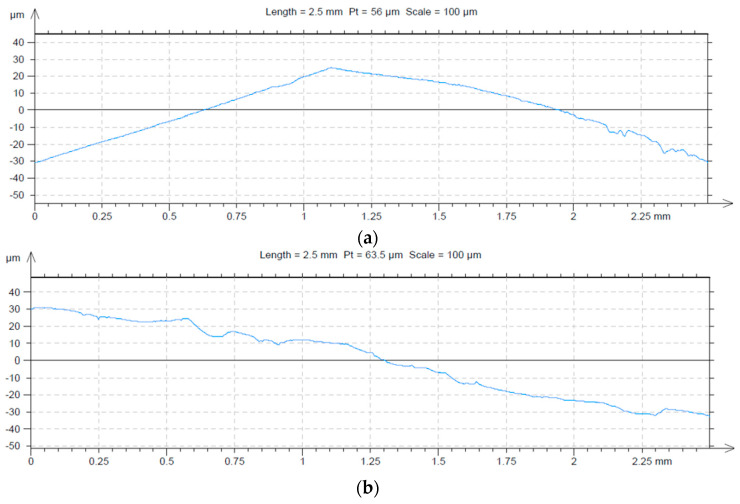
Profile images of damaged surfaces: (**a**) original contact based on copper coated with silver, (**b**) composite material based on CuCr1W5.

After the testing process of the contacts, the damaged surfaces of the samples were measured using a Surtronic 25 profilometer, Taylor Hobson (Leicester, United Kingdom, 2007), with the following characteristics: calibration limit—300 µm, resolution—0.01 µm, examination length—0.25–25 mm, examination speed—1 mm/s, and computer connection RS232. The profile images are presented in Figure 10.

## 3. Results and Discussions

**Figure 11:**
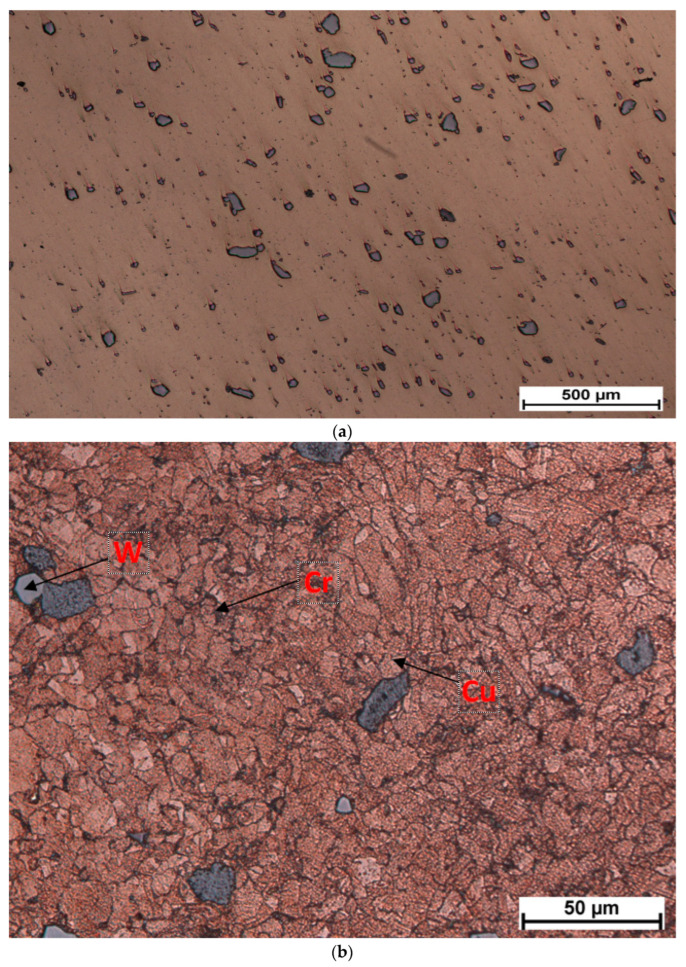
Microstructure of sample: (**a**) non etched (just polished) 75×, (**b**) etched (cupric chloride) 750×.

The development of a new contact based on an active element from the composite material CuCr1W5 has been performed, starting from reports [1,2], related to the level of malfunction of the electrical contacts embedded in reactive/capacitive load breakers. The development of a new contact based on the composite material CuCr1W5 has been performed using an MHH process for sintering. The results related to the microstructure of the sample are presented in Figure 11. According to Figure 11b, the grain size of tungsten is 25 µm, the chromium grain size is below 5 µm, and the grain size of the copper matrix is also below 25 µm. The structure of composite material seems to be uniformly distributed.

Regarding the soldering of the sintered composite material and the copper contact body, and the low interval of the soldering temperature (217–228 °C for SAC305 solder) is not affected by the thermal runaway phenomenon, but the instability of the microwave soldering process must be addressed. The instability of the process can be observed in the balance of power during microwave heating. After 20 s of heating, the reflected microwave power P_ref_ [W] oscillates between 66 W and 79 W, which represents more than 10% of the total injected power. For small applications, this level of reflected power does not represent an issue; but, for multiple soldering processes (wide scale productions similar to industrial processing) in the same reaction oven, the loss of microwave power will lead to a low quality of the joints.

**Figure 12:**
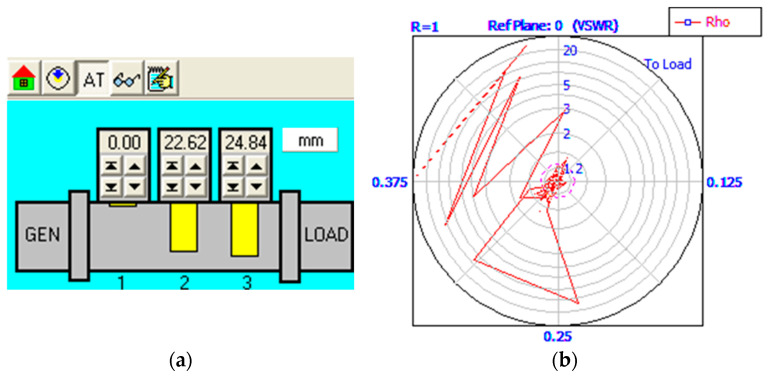
Matching load impedance of assembly CuCr1W5—dolomite to the microwave generator during the soldering process: (**a**) stub tuners position, (**b**) absorbed/reflected microwave (courtesy Homer Software V5.2).

In addition to Figure 7 and according to Figure 12, the matching load impedance of the assembly metal sample-susceptor is difficult to achieve due to the presence of the metal in the microwave field. However, the circuit impedance remains at low values mainly due to the scenario with the position of the first stub tuner to null (d_1_ = 0 mm), which allows the passing of microwaves from the generator to the load. In addition, the calculated positions for the other stub tuners (d_2_ = 22.62 mm, d_3_ = 24.84 mm) do not allow the reflected microwave to pass the waveguide WR340 back to the microwave generator.

The testing of new contact functionality and performance has been realized using an experimental device. After 111,237 cycles, the system reported an error T-phase on the breaking cycle. This led to the conclusion that the contact suffered during the temporary welding process and that breaking was no longer available, for a short time, for all three phases.

**Figure 13:**
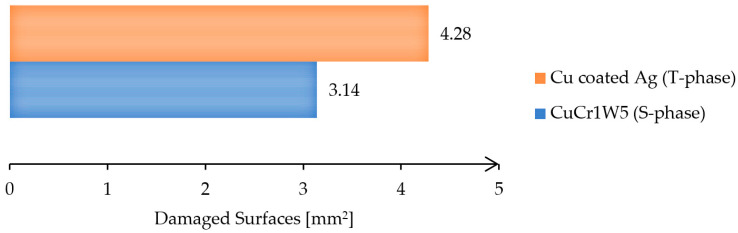
Areas of damaged surfaces of the contacts.

After suspending the testing process, the electrical contacts have been analyzed under a microscope to evaluate the joint quality and to identify the defects. Figure 13 presents a comparative analysis of the damaged area for the T-phase and S-phase contacts.

**Figure 14:**
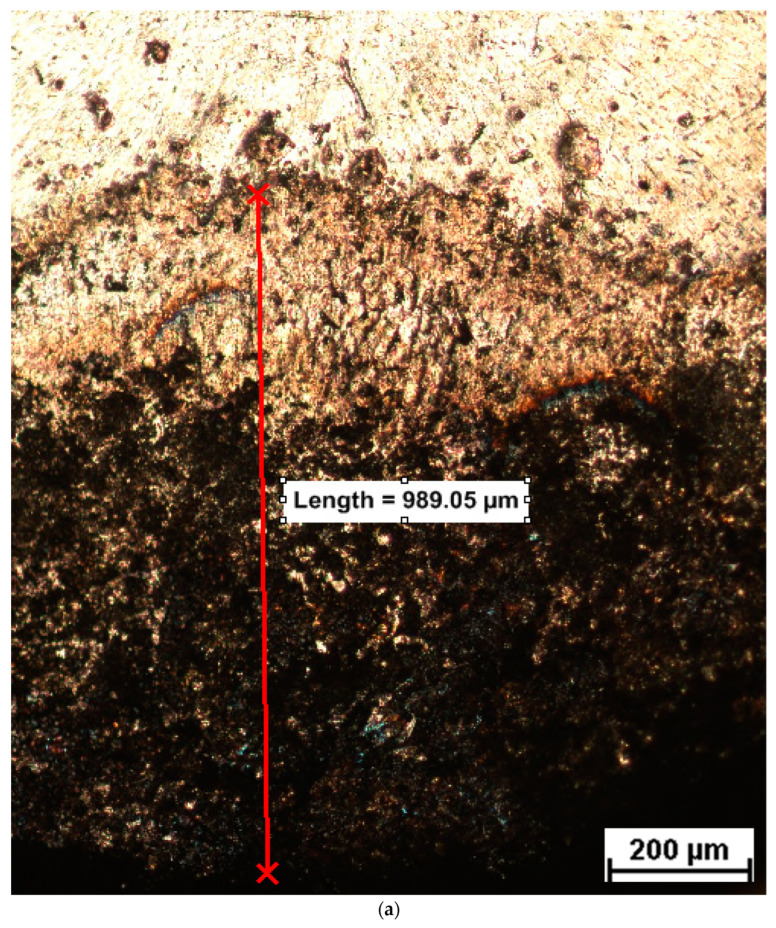

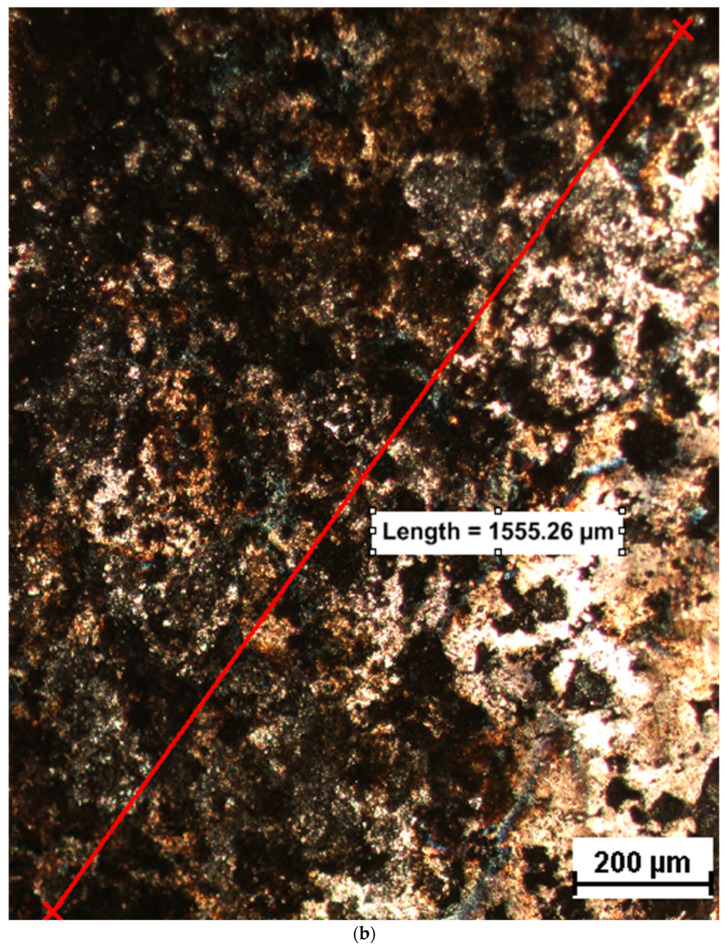
Surface structure analysis: (**a**) S-phase contact for composite material based on CuCr1W5, (**b**) T-phase contact for original contact based on copper coated with silver.

The chart presented in Figure 13 shows that the damaged area of the composite material based on CuCr1W5 is around 3.14 mm^2^, being 27% smaller than the damaged area, around 4.28 mm^2^, recorded for the original contact based on copper coated with silver. This can be explained by the properties of tungsten (a high melting point around 3422 °C and a high thermal conductivity) related to its level of degradation at a high temperature as well as its behavior under high AC currents. This behavior under high AC currents consists, of allowing it to withstand extreme heat without melting or deforming, is due to tungsten’s high melting point. In addition, tungsten has a relatively low electrical resistance, meaning it can carry large currents efficiently but without significant erosion or degradation. On the other hand, according to Figure 14a, the new contact was affected mainly at the edge of the surface, which can be explained by the lack of contact on the entire surface. An analysis of the structure on the surface of the contacts used in the experimental program is presented in Figure 14.

**Figure 15:**
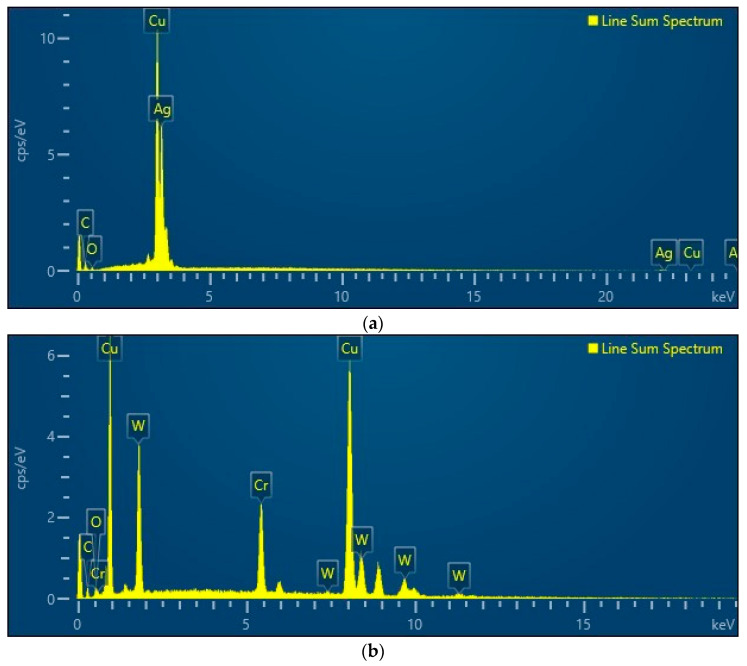
EDS analysis: (**a**) original contact based on copper coated with silver, (**b**) new contact from composite material based on CuCr1W5.

The macroscopic examination of Figure 14b reveals that the electrical contact surface experienced electro-erosion, likely due to electric arcs forming during the closing or opening of the contacts. The effect is double: a thermal stress producing oxidation on about 20% of the surface, including oxides of copper, tungsten, and chromium, indicating exposure to an open-air environment, and a material removal, with excavation depths ranging from 21 to 56 µm, possibly caused by imperfections like microcracks. Thermal stress and its effect of thermal expansion produced oxidation on about 20% of the surface, including oxides of copper, tungsten, and chromium, indicating exposure to an open-air environment. The presence of the oxide types has been determined using an EDS analysis performed using TESCAN VEGA LMU equipment produced by TESCAN Brno s.r.o (Brno, Czech Republic, 2023). The results are presented in Figure 15. Regarding the original contact, the access of oxygen to copper is in the areas in which the silver is removed by extremely fast vaporization under electric discharge. But the entire silver layer is not removed by vaporization, and oxides of silver are present even in the affected area. The authors know that the potential of copper to oxidize is higher, but silver has the potential at the highest exposure to oxygen.

Regarding the composite material, the order of potential of oxidation is: copper, followed by chromium and tungsten. Oxygen is present and it has access to copper and chromium under high temperatures, producing a large quantity of copper oxides and chromium oxides in the affected area by discharge.

## 4. Conclusions

The lifespan of electrical contacts can be extended by using composite materials in relay contacts. The composite was sintered using hybrid microwave heating with dolomite-based susceptors, as direct microwave heating is challenging due to the low absorbance of metal. Simulations determined that the optimal microwave settings to achieve a sintering temperature was 1012 °C with 623.38 W of power. The sintering process took 65 min in total, which is much faster than conventional methods.

The composite material was then soldered to a copper contact body, with simulations again guiding the process. A soldering temperature of 231 °C was reached in 55 s using 475 W of power, making it more efficient than traditional soldering.

The new electrical contact was tested according to ICE standards, using a device designed to handle a 5 kVAr capacitive load. After 111,237 cycles, an error was detected in the T-phase due to contact welding.

## Figures and Tables

**Table 1:** Main characteristics of elemental powders.

Parameter/Powder	Cu	Cr	W
Apparent Density	2.30–2.50 g/cm^3^	7.14 g/cm^3^	18.5 g/cm^3^
Purity	99.7%	99.8%	99.1%
Mesh size	180–212 µm	70 µm	80 µm
Grain shape	dendritic	irregular	polyendric

**Table 2:** Technological parameters of mechanical alloying process of W–Cu–Cr powders.

Parameter	Value
Bowl volume	250 mL
Ball diameter	10 mm
Ball material	stainless steel
Alloying environment	dry inert gas (Ar)
Ratio material/balls	1/2
Rotation speed	5.83 1/s on primary drive 8.33 1/s on bowl
Alloying time	5 h

**Table 3:** Heat properties and coefficients of materials.

Material	k (W/mk)	α (10^−6^ m/m°C)	c (kJ/kgK)
Aluminum	231	22	1042
Dolomite	5.3	4.6	0.92
CuCr1W5	398	17	390

**Table 4:** Microhardness of composite material.

Test Parameters	Test 1	Test 2	Test 3	Average
Vickers method Tip angle 136° Load 4.9 N (HV0.5) Dwell time 15 s	45.4	51.9	48.5	48.6

## Data Availability

The original contributions presented in the study are included in the article, further inquiries can be directed to the corresponding author.
